# Obituary

**Published:** 1909-03-15

**Authors:** 


### OBITUARY.

### DR. LUIS LANE DUNBAR.

Died, at his home in Belvedere, Cal., on Wednesday morning, December 30th, 1908, Luis Lane Dunbar, D.D.S., in his sixtieth year. While Dr. Dunbar had not been in robust health for some months before, the end came as a great surprise and very quickly, as he had devoted the day before to work in his office and had not complained of anything unusual up to a few minutes before his death.

He was born in Evansville, Indiana, September 1st, 1849, and in 1863 went to San Francisco, where he attended the public schools, and then St. Mary's College. He later went to Ohio, being graduated in dentistry from the Ohio College of Dental Surgery in 1874, returning then to California.

Dr. Dunbar was one of the early practitioners of dentistry of that state, having located in San Francisco thirty-five years ago. He early reached a prominent and enviable position in the profession, and for a great many years was an active member in different dental societies and educational work.

Aside from his chosen life-work of dental practice which engaged so much of his time and attention, he was no less devoted to the Dental Department of the University of California, becoming identified with it at the time of its organization in 1882. He gave freely of both his time and energy in its interest for a great many years, and kept in touch with it, enjoying an honored position on its staff to the time of his death. He served it in different capacities, first as clinical instructor, 1882.84, then as professor of pathology and therapeutics, in 1885, and from 1888 to 1889 as professor of operative dentistry and dental histology He was re-elected dean of the college each year from 1889 to 1899. From the last named date to the end of his life he occupied the position of emeritus professor of operative dentistry.

He was a member of the California State Dental Association, of the Free Masons, and a life-member of Delta Sigma Delta Fraternity.

In 1874, he married Miss Jennie McLaughlin, who survives him, together with their son, Stewart Boyd Dunbar.—February COSMOS.

